# Genomic mid-range inhomogeneity correlates with an abundance of RNA secondary structures

**DOI:** 10.1186/1471-2164-9-284

**Published:** 2008-06-12

**Authors:** Jason M Bechtel, Thomas Wittenschlaeger, Trisha Dwyer, Jun Song, Sasi Arunachalam, Sadeesh K Ramakrishnan, Samuel Shepard, Alexei Fedorov

**Affiliations:** 1 Program in Bioinformatics and Proteomics/Genomics, University of Toledo Health Science Campus, Toledo, OH 43614, USA; 2 Dept. of Physiology and Pharmacology, University of Toledo Health Science Campus, Toledo, OH 43614, USA; 3 Dept. of Neuroscience, University of Toledo Health Science Campus, Toledo, OH 43614, USA; 4 Department of Cardiovascular and Metabolic Diseases, University of Toledo Health Science Campus, Toledo, OH 43614, USA; 5 Dept. of Medicine, University of Toledo Health Science Campus, Toledo, OH 43614, USA; 6 Department of Biological Sciences, Bowling Green State University, Bowling Green, OH 43403, USA; 7 Luye Pharmaceutical LTD, Rm1107, Zhubang 2000 Business Center, Chaoyang District, Beijing 100025, PR China

## Abstract

**Background:**

Genomes possess different levels of non-randomness, in particular, an inhomogeneity in their nucleotide composition. Inhomogeneity is manifest from the short-range where neighboring nucleotides influence the choice of base at a site, to the long-range, commonly known as isochores, where a particular base composition can span millions of nucleotides. A separate genomic issue that has yet to be thoroughly elucidated is the role that RNA secondary structure (SS) plays in gene expression.

**Results:**

We present novel data and approaches that show that a mid-range inhomogeneity (~30 to 1000 nt) not only exists in mammalian genomes but is also significantly associated with strong RNA SS. A whole-genome bioinformatics investigation of local SS in a set of 11,315 non-redundant human pre-mRNA sequences has been carried out. Four distinct components of these molecules (5'-UTRs, exons, introns and 3'-UTRs) were considered separately, since they differ in overall nucleotide composition, sequence motifs and periodicities. For each pre-mRNA component, the abundance of strong local SS (< -25 kcal/mol) was a factor of two to ten greater than a random expectation model. The randomization process preserves the short-range inhomogeneity of the corresponding natural sequences, thus, eliminating short-range signals as possible contributors to any observed phenomena.

**Conclusion:**

We demonstrate that the excess of strong local SS in pre-mRNAs is linked to the little explored phenomenon of genomic mid-range inhomogeneity (MRI). MRI is an interdependence between nucleotide choice and base composition over a distance of 20–1000 nt. Additionally, we have created a public computational resource to support further study of genomic MRI.

## Background

### RNA secondary structures

Secondary structures (SS) are crucial elements for the biosynthesis and/or correct action of non-coding RNAs in mammals and other eukaryotes. Moreover, they are key regulators in the function and turnover of mRNA molecules. SS in pre-mRNAs regulate the splicing process [1-3]. In mature mRNAs, SS located in 5'-untranslated regions (5'-UTRs) signal for translational control [4,5] and those located in 3'-untranslated regions (3'-UTRs) regulate sub-cellular localization and stability [6-8]. SS located inside protein-coding sequences could play a role in translational speed and stability [9,10].

Prior studies of the strength of computer-predicted SS in mRNA have had conflicting conclusions [11,12]. More importantly, these studies did not investigate the abundance of SS and considered only coding sequences. This spurred us to perform a bioinformatics investigation into the abundance of SS throughout mammalian genomes. Our results show that the existence of many energetically-strong SS is associated with the phenomenon of global mid-range inhomogeneity (MRI), manifest as nucleotide compositional relationships at a scale of 20 to 1000 bases throughout the genome. MRI appears as a strong tendency for the clustering of particular bases (e.g. C and G nucleotides, or G and A nucleotides) inside short regions of genomic sequences. This paper provides new approaches and tools to gain insights into this form of genomic inhomogeneity.

### Short-range inhomogeneity

It is well established that the particular base (A, G, C, or T) that appears in a given position of a genomic sequence significantly depends upon the nearest bases surrounding its position [13,14]. Consequently, the frequency (F) of a dinucleotide *XY *is often not equal to the product of the individual frequencies of nucleotides *X *and *Y *(F_*XY *_≠ F_X _*F_*Y*_). The highest interdependence of base frequencies is always observed for adjacent nucleotides. The ratio (F_XY_/(F_X _*F_Y_)) for adjacent bases *X *and *Y *is known as a "genomic signature" [14]. Genomic signatures as low as 0.22 (for the CG dinucleotide in mouse) and as high as 1.75 (for the GC dinucleotide in *Campylobacter jejuni*) have been recorded [15]. The interdependence of base frequencies sharply drops with increasing distance. When the distance between nucleotides *X *and *Y *is more than six bases, their occurrence interdependency becomes negligible. Here, we refer to this type of interdependency between nucleotides separated from each other by a few positions as *short-range inhomogeneity *(SRI).

### Long-range inhomogeneity

Also well recognized are long-range interdependencies in nucleotide frequencies on a scale of up to millions of bases, known as genomic *isochores *[16]. It has been shown that isochores can be generally categorized according to their level of G+C content. Isochores defined by G+C content correspond to many other genomic phenomena. GC-rich isochores replicate later in S-phase, contain higher concentrations of genes, and have genes with shorter introns and untranslated regions. Moreover, GC-rich isochores tend to have an "open" chromatin structure and thus have higher rates of transcription [17]. Higher G+C content isochores also experience higher recombination rates – perhaps lending support to the notion that higher recombination rates led to the creation of isochores through biased gene conversion [18]. While the evolution and maintenance of isochores is subject to debate, their presence is indeed evidence of existing interdependencies in nucleotide composition on the scale of tens of thousands to millions of nucleotides. We will refer to this form of non-randomness in genomic nucleotide composition as *long-range inhomogeneity*.

### Mid-range inhomogeneity

The compositional non-randomness between the two extremes described above we call *mid-range inhomogeneity *or MRI. MRI has yet to be thoroughly investigated. The only well-known manifestation of mid-range inhomogeneity is CpG islands. Most attempts to define CpG islands set hard requirements for region size (at least 200 or 500 bases long), G+C content (> 50% or 55%), and CpG observed/expected ratio (> 0.6 or 0.65) [[19,20], respectively]. CpG-islands are found near 60% of human genes, including all housekeeping genes and about half of the tissue-specific genes [21]. Here we demonstrate that MRI can be observed for regions from 30–1000 bp and is significant not only for G+C content but for other nucleotide pairings (A+G and G+T) as well as for the individual bases.

## Results

### Analysis of strong local SS within pre-mRNAs

Distinct parts of mRNAs and introns have large variations in nucleotide composition (from 35% to 60% of GC-content, see Table 1). Due to this difference the analyses of SS distribution were performed on four separate regions: GC-rich 5'-UTR regions, GC-poor introns and 3'-UTR regions, and intermediate GC-content protein-coding regions of mRNAs. In addition, vertebrate and invertebrate species have considerable variations in their mRNA nucleotide composition. Within the mammalian class, however, the variation in GC-content is negligible (Table 1). For this reason we demonstrate only results for human sequences, although the observed trends are applicable to all mammals.

**Table 1:** Percentage of GC-composition in different regions of pre-mRNA for diverse animal species.

SPECIES	% GC-content			
	5'-UTRs	CDS	3'-UTRs	Introns

Human	60%	52%	44%	41%
Mouse	59	52	44	43
Cow	60	54	44	43
Chicken	57	51	41	41
Zebrafish	45	50	37	35
Drosophila	45	54	36	40

**Figure 1:**
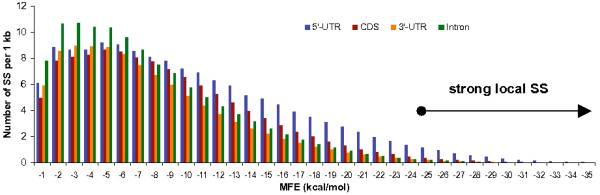
**Distribution of local SS with respect to folding energy in mRNA components and introns**. Number of structures was measured within 1 kcal/mol intervals and normalized by 1,000 nucleotides of analyzed sequences.

We begin by looking at SS created by the interactions of nucleotides less than 50 bases apart (local SS). Prediction of local SS is more reliable than prediction of global RNA structures, which can span hundreds of nucleotides [22]. We examined these structures in 11,315 non-redundant human gene sequences (see Methods section) and calculated their strengths using the RNALfold program of the Vienna RNA package [23]. Figure 1 illustrates the distribution of local SS according to folding strength in distinct parts of mRNAs and introns. We concentrate our study on the stronger secondary structures. Such strong local SS could withstand competition with the many RNA-binding proteins that cover mRNAs and, thus, are more likely to persist *in vivo*. Indeed, hairpin structures with an mfe of -30 kcal/mol situated close to the mRNA cap significantly impede ribosome scanning, while hairpin structures with an mfe upwards of -50 kcal/mol inhibit elongation [5]. Strong secondary structures are also required for the regulation of splicing. Kishore and Stamm showed that the 18 nt-long HBII-52 snoRNA antisense element interacts with a pre-mRNA segment with an mfe of -27.6 kcal/mol and thereby determines the fate of alternative splicing in the serotonin receptor gene [24]. Finally, a majority of human miRNA genes from the microRNAdb [25] have strong interaction energies (< -25 kcal/mol) with dozens of targets within mRNAs. Thus, "strong" local SS is defined as having a minimum free energy (mfe) value of less than or equal to -25 kcal/mol, so as to encompass all known functional local SS.

### Analysis of strong local SS in randomized sequences

To evaluate the abundance of local SS, one must compare their prevalence in naturally occurring mRNAs with their levels in reference sequences having no selection for SS. In most research, reference sequences are randomly generated to have nucleotide compositions approximating those of the naturally occurring mRNAs. In order to properly compare local SS in natural and randomized sequences one needs to preserve short-range inhomogeneity (SRI), as discussed previously by Workman and Krogh [12].

Thus, to most accurately preserve SRI in randomized sequences we created a public resource for generating randomized sequences while taking into account the SRI of a given set of natural sequences. Our algorithm can take into account not only relative *di*nucleotide frequencies, but also frequencies of longer oligonucleotides (up to 9-mers). We first applied our SRI-analyzer program (see Methods section) [26] to a set of natural mRNA sequences to obtain their oligonucleotide composition, shown in Table 2. Our second program, SRI-generator [26], then uses these oligonucleotide frequency tables to generate random sequences with approximately the same oligonucleotide distribution as the natural sequences but without any similarity in their sequence alignments. Table 2 demonstrates the oligonucleotide frequencies for human 5'-UTRs and two independent SRI-generated sets of sequences. Notably, the oligonucleotide compositions of the SRI-generated sequences are very close to those of the natural sequences, with only small fluctuations due to the inherently random nature of the sequence generation process [see Additional file 1]. Pseudocode and further explanation of SRI-generator is provided for the reader as an additional file [see Additional file 2].

**Table 2:** Excerpt from oligonucleotide frequency table for 5'-UTRs of 11,315 human genes and two SRI-generated counterparts. The entire dataset is presented in Additional file 1.

Oligonucleotide	Human 5'-UTRs	Random 1 SRI-generated	Random 2 SRI-generated
A	0.203	0.203	0.203
T	0.201	0.201	0.2
C	0.292	0.294	0.293
G	0.303	0.303	0.303
AA	0.0506	0.0503	0.0506
AT	0.0323	0.0323	0.0318
AC	0.0438	0.0437	0.0438
AG	0.0771	0.0767	0.077
TA	0.0258	0.0257	0.0256
TT	0.0511	0.051	0.0508
TC	0.0593	0.0591	0.0588
TG	0.0653	0.0651	0.0651
CA	0.0608	0.0609	0.0608
CT	0.0726	0.0726	0.0725
CC	0.0965	0.0973	0.0968
CG	0.0627	0.0627	0.0629
GA	0.0658	0.0661	0.0662
GT	0.0453	0.045	0.0452
GC	0.0932	0.0934	0.0936
GG	0.0977	0.098	0.0984
...	...	...	...
ACAA	0.002558	0.002507	0.002544
ACAT	0.002046	0.00205	0.002057
ACAC	0.002775	0.002731	0.0028
ACAG	0.004442	0.004362	0.00442
ACTA	0.001396	0.001362	0.001388
ACTT	0.002893	0.002899	0.002881
ACTC	0.003028	0.002958	0.003063
ACTG	0.003961	0.003912	0.003954
...	...	...	...

**Figure 2:**
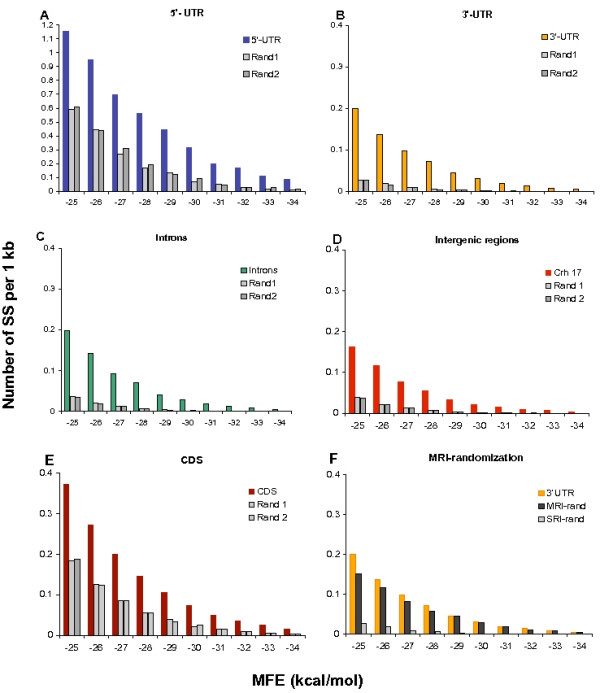
**Distribution of strong local SS with respect to folding energy in mRNAs and genomic sequences**. Number of structures was measured within 1 kcal/mol intervals and normalized by 1,000 nucleotides of analyzed sequences. (**A**) 5'-UTRs (blue) and two independent SRI-generated sequences (gray); (**B**) 3'-UTRs (yellow) and two independent SRI-generated sequences (gray); (**C**) introns (green) and two independent SRI-generated sequences (gray); (**D**) intergenic regions from chromosome 17 (red) and two independent SRI-generated sequences (gray); (**E**) CDS (burgundy) and two independent CDS-generated sequences (gray); (**F**) 3'-UTRs (yellow), random MRI-generated counterpart sequences (black), and random SRI-generated counterpart sequences (gray).

Figure 2A–C demonstrate the distribution of strong local SS in human 5'-UTR, 3'-UTR and intronic sequences and in their corresponding randomized counterpart sequences. Prediction of local SS was computed on a non-redundant sample of 11,315 human genes (see M&M) by the RNALfold program [23]. All SRI-randomization was performed based on the tetramer frequency tables of the corresponding natural sequences. Tetramers reflect almost all short-range non-randomness since the major influence on SRI comes from adjacent bases [27]. Remarkably, the number of local SS in the natural sequences exceeds the number of structures of the same strength in the random sequences by a factor of at least 2 to 10. Because we processed eleven thousand genes, the significance of this difference is unquestionable: the chi-square goodness-of-fit test gives a p-value less than 10^-200^. Having observed the difference between introns and their SRI-generated counterparts, we also examined sequences from intergenic regions (located between protein-coding genes and having an overall nucleotide composition similar to that of introns) and detected the same trend (Figure 2D). Moreover, the abundance of SS in natural sequences has no relation to genomic repetitive elements. Masking all human repeats with the RepeatMasker program [28] even mildly enhanced the difference in strong local SS between natural and SRI-generated sequences [see Additional file 3]. This observation simply reflects the fact that DNA repeats do not have an excess of strong SS, although some repeats have a distinct oligonucleotide composition and are enriched by C/G bases (e.g. Alu family).

Protein-coding sequences (CDS) contain a profound 3 nt periodicity and other non-randomness associated with translational properties [29]. All of this information would be lost in SRI-generated sequences. To overcome this problem we created CDS-generator, a public resource for the randomization of protein-coding sequences [26]. CDS-generator changes only the variable nucleotides in the third codon position, which do not change the coded amino acids. Additionally, CDS-generator maintains the codon and dicodon biases of a given set of natural coding sequences. Thus, randomization by CDS-generator is much weaker than randomization by SRI-generator since it retains > 70% sequence identity between the natural and random sequences. On the other hand, maintaining a considerable level of sequence identity is useful because it preserves the major periodicity characteristics of the source coding sequences. Figure 2E demonstrates that natural coding sequences have twice the number of strong local SS as randomized sequences obtained by CDS-generator. The chi-square test confirms that the difference is statistically significant (p < 10^-200^).

### Mid-range inhomogeneity in natural genomic sequences

**Figure 3:**
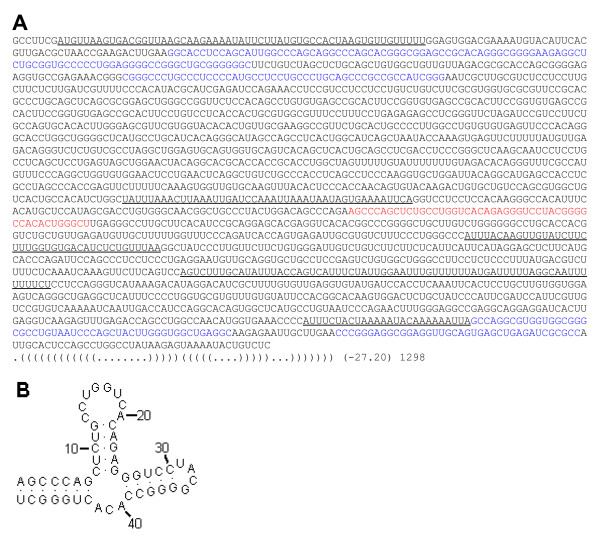
**Example of a strong local SS in the 3'-UTR of the human KIAA1751 gene **[GenBank:NM_001080484]. (**A**) Nucleotide sequence of the entire 3'-UTR region in which a segment exemplifying a strong local SS (mfe = -27.2 kcal/mol) is shown in red and its schematic base-pairing is shown in dot-bracket notation [23] below the sequence. Other GC-rich regions are highlighted in blue and GC-poor regions are underlined. (**B**) 2-D representation of this strong SS.

To understand the observed abundance of strong local SS in mRNAs, we examined dozens of these structures in natural sequences, a typical example of which is shown in Figure 3. This structure, from a human 3'-UTR of the *KIAA1751 *gene, has an mfe of -27.2 kcal/mol and represents a hairpin stem-loop configuration. The sequence of this SS is GC-rich (67%) and is neighbored by several other alternating short AT-rich and GC-rich regions, as highlighted in Figure 3A. In contrast, such frequent alternation in GC-composition is practically absent in SRI-generated sequences. Statistical examination revealed that strong local SS with mfe values in the range -25 to -30 kcal/mol in human mRNAs have a mean GC-composition of 70%, which is much higher than the average GC-composition of the mRNA, introns, or intergenic regions presented (Table 1). The observed GC-enrichment within strong local SS can be explained by thermodynamics (G-C base pairs are about twice as strong as A-T pairs) and by combinatorics (random base-pairing is more frequent in GC-rich strands than when GC-composition is around 50%). These notions have led to the hypothesis that natural sequences have a profound mid-range inhomogeneity, that is, they are enriched by short GC-rich regions (30–100 nt) alternating with adjacent AT-rich regions. In other words, we theorize the non-random clustering of G/C and A/T bases on the scale of ~50 nucleotides and the over-abundance of such clusters in natural sequences.

**Figure 4:**
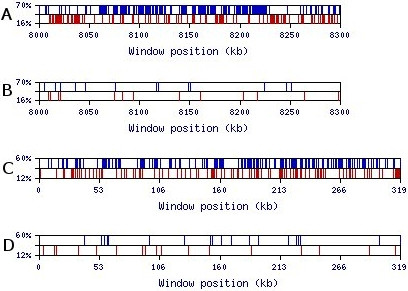
**Visualization of MRI-analyzer output for GC-composition of two 300 kb samples using a 50-nt window**. Upper and lower thresholds are specified on the y-axis as a percentage of the window size. (**A**) A sequential sample of human 3'-UTRs from chromosomes 1 and 2 (EID ids 1745_NT_004487 through 2327_NT_022184); (**B**) a random SRI-generated set based on the tetramer oligonucleotide frequency table of 11,315 human 3'-UTR sequences. (**C**) The 319 kb sequence of the first extra-large intron of the *DMD *gene; (**D**) a random SRI-generated set based on the tetramer oligonucleotide frequency table of the first intron of the *DMD *gene.

**Figure 5:**
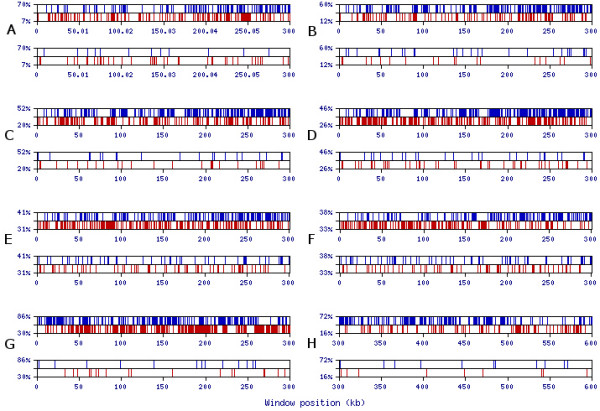
**Comparison of MRI-analyses of GC-content for various window sizes and genomic contexts**. **(A-F) **The 319 kb sequence of the first intron from the *DMD *gene, and its SRI-generated counterpart, analyzed for optimal visual contrast over a range of window sizes (30, 50, 100, 200, 500, 1000) (cf. Figures 4 and 7); **(G) **The first 300 kb of a sample of human 5'-UTRs and its SRI-generated counterpart using a window size of 50 nt; **(H) **The 300 kb subset from a sample of intergenic sequences from human chromosome 17 and a corresponding SRI-generated sequence using a window size of 50 nt.

**Figure 6:**
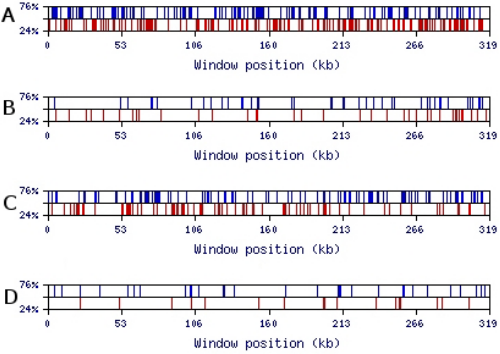
**Visualization of MRI-analyzeroutput for AG- and GT-compositions of 319 kb sequence of the first intron of the DMD gene using a 50 nt window**. Upper and lower thresholds are specified on the y-axis as a percentage of the window size. (**A**) AG-rich and AG-poor regions of the *DMD *intron; (**B**) AG-rich and AG-poor regions of the corresponding random SRI-generated set based on the tetramer oligonucleotide frequency table of the *DMD *intron; (**C**) GT-rich and GT-poor regions of the *DMD *intron; (**D**) GT-rich and GT-poor regions of the corresponding random SRI-generated set based on the tetramer oligonucleotide frequency table of the *DMD *intron.

**Figure 7:**
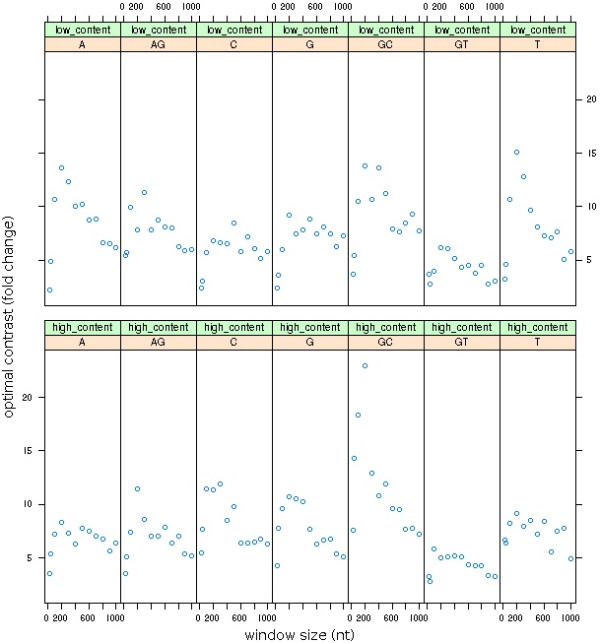
**Optimal contrasts for all content types over a range of window sizes**. This figure is the "XY conditioning plot" (from the program Rcmdr 1.2) of the optimal contrasts (see text) for regions of high and low content for all seven possible content types over a range of window sizes (30, 50, 100, 200, 300, ... 1000). The sample sequence is the 319 kb first intron from the *DMD *gene. The SRI-generated counterpart is constructed from the tetramer frequency table derived from the intron.

To test this hypothesis we created a program named MRI-analyzer [26]. This program scans input sequences with a mid-range window size (the default, utilized for the results presented, is a 50 nt window). When the GC-content of the sequence in this window reaches the upper threshold, MRI-analyzer generates a blue top spike on the output graph (Figure 4). Similarly, when the GC-content of the window reaches the lower threshold, MRI-analyzer generates a red bottom spike. The upper and lower thresholds are flexible parameters defined by the user. MRI-analyzer output for natural and SRI-generated 3'-UTR and intronic sequences is shown in Figure 4. Here, the graph clearly shows a 35-fold enrichment of GC-rich (≥ 70%) 50 nt-long regions in natural 3'-UTR sequences over the randomized SRI-generated sequences. For the 319 kb single extra-large intron in Figure 4C–D, GC-rich regions are enriched by a factor of 14. Similar to the results shown in Figure 4, we observed comparable contrasts in GC-rich and GC-poor regions for 5'-UTR, intronic, and intergenic sequences for a wide range of scanning window sizes (30–1000 nt) (Figure 5). Additionally, MRI was generally observed with other base combinations and single bases (Figure 5). An example of the MRI pattern for GA- and CT-rich regions is shown in Figures 6A and 6B; GT- and AC-rich regions are presented in Figures 6C and 6D. The enrichment factor can be considered as the contrast between real and randomized sequences. We thus use the term "contrast" to refer to the ratio of the number of content-rich regions in a real sequence to the number in its SRI-generated counterpart. "Optimal contrast" is defined as the highest contrast observed over all thresholds for a given content type (for example, GC-content) and window size. Since we do not yet know the properties of mid-range inhomogeneity in the human genome, we probed for the optimal contrast by repeating the above analysis for all thresholds starting from one standard deviation above (and below) the mean GC-content and proceeding until the number of GC-rich (or GC-poor) regions decreases to about ten. Figure 7 shows the distribution of optimal contrasts for all content types and a range of window sizes for the longest first intron of the *DMD *(dystrophin) gene.

### Association of MRI with the over-abundance of strong local SS

Finally, we created a program named MRI-generator [26] for obtaining random sequences having the same oligonucleotide composition and also the same MRI pattern in GC-composition as a specified set of natural sequences. This program works by producing an excessively long SRI-generated sequence and then discarding segments with intermediate GC-content to obtain the desired pattern of GC-rich and CG-poor regions. Thus, the output sequence from MRI-generator has a Genomic-MRI pattern of GC-rich and GC-poor regions very similar to that of the natural sequence.

Comparison of natural sequences with their MRI-generated counterparts for each genomic sequence category (5'-UTRs, 3'-UTRs, introns, intergenic regions, and CDS) shows that they each have approximately the same number (5–10% difference) of strong local SS, as illustrated in Figure 2F for 3'-UTRs. This finding supports the conclusion that the abundance of strong SS in all parts of the mammalian genome (mRNA, introns, intergenic regions) is associated with the MRI of these sequences.

### DNA repetitive elements and genomic MRI

Even though human interspersed repeats do not show an excess of strong SS as discussed above, they do influence the patterns of MRI [see Additional file 4]. The figure in additional file 4 illustrates the MRI patterns of the extra-large first intron of the *DMD *gene (see Figure 4) after masking its repetitive elements by RepeatMasker. Unsurprisingly, the number of MRI regions in the masked sequence is a fraction of those in its non-masked counterpart. The masked sequence contains 41% N's instead of A, G, C, or T bases. The current version of MRI-analyzer skips a window containing any non-A, G, C, or T character. For a proper comparison of MRI patterns in a masked sequence, one should compare it to the SRI-generated random sequence based on the masked sequence. Such random sequences contain the same number of N's at exactly at the same positions as the natural masked sequence. The figure in Additional file 4 demonstrates that the masked sequence of the first *DMD *intron has 3 to 12 times the number of MRI peaks compared to its random counterpart. This particular example with the *DMD *intron presents an AT-rich sequence (67% of A+T), which is typical for extra-large introns [17]. Accordingly, we set the upper threshold for GC-composition to 60% in studying this sequence. Under such conditions, we observe GC-rich MRI peaks overlapping various portions of Alu-repeats. This overlapping of MRI regions with repetitive elements seems to depend on the threshold used and the G+C-composition of the region under analysis.

## Discussion

We have demonstrated an association between MRI in GC composition and the abundance of strong SS in genomic sequences. There are at least two possible interpretations of these results. First, one can argue that MRI causes the abundance of strong SS. The second possibility is that selection for strong SS was the reason for the appearance of MRI. Both views have merit and we thus include a discussion of the supporting evidence.

Central to this discussion is the observation that MRI exists not only in mRNA sequences but also in introns and intergenic regions. If selection were limited to transcripts or to mature mRNAs, there would be no way for evolution to directly drive the creation of SS in non-transcribed regions. This would leave MRI in GC composition as a potential mediator of strong SS enrichment. However, some experimental evidence suggests that much more of the genome is transcribed than previously thought [30]. It has also been suggested for some time that SS play a role in the initiation of recombination. This theory predicts positive selection for SS throughout genomes and especially within introns and intergenic regions [31-33]. Moreover, studies of coding sequences in mammals have found that synonymous substitutions tend to increase the strength of SS and regulate mRNA stability [34-36]. Thus, SS could have emerged first due to selection for DNA hairpins to facilitate homologous recombination and for stable mRNA SS signals, yielding MRI in GC content as a by-product. On the other hand, MRI is also observed for AG- and GT-content as well as for the individual nucleotides (see Figures 6 and 7), so it is also possible that selection for MRI is a fundamental force driving genome organization and composition.

It is of special interest to investigate possible biological roles for MRI in the structural and functional organization of mammalian genomes. To address this important issue, we have studied 3.3 million point mutations occurring over the last 10 million years in humans and over 3.9 million SNPs in the MRI-regions and outside them. These results will be detailed in our next publication (under preparation). Based on the preliminary results of these investigations, we can state that MRI patterns are formed by a combination of processes. Some patterns (e.g. A+T-rich regions) are like cellular automata, based on non-selection biases in nucleotide changes at genomic regions with specific base compositions, while other patterns are formed by a strong fixation bias (presumably positive selection of functional regions) that preserve particular base enrichments in corresponding regions (e.g. G+C-, purine-, and pyrimidine-rich). These forces drive mid-range non-randomness, shaping the human genome and potentially imparting additional layers of organizational complexity.

Indeed, an important feature of the human genome is that its vast array of genes is differentially expressed in hundreds of different cell types and subtypes. Moreover, at different stages of development and in response to diverse extracellular stimuli, gene expression must be finely tuned. To perform the enormous task of creating a human body composed of trillions of cells, the genome must contain a vast number of signals for gene regulation, the majority of which have yet to be discovered. We hypothesize that MRI could represent a novel class of genomic signals, based on overall composition and clustering of nucleotides rather than particular sequence motifs. To facilitate the testing of this hypothesis, we created a free, public Internet resource called "Genomic MRI" that allows one to run all programs described here without any programming knowledge. Additionally, all of these programs are freely available for downloading and off-line usage, primarily for computational biologists.

## Methods

The programs SRI-analyzer, SRI-generator, MRI-analyzer, MRI-generator, and CDS-generator are available via our website. A link to the current location of the website will be maintained at our departmental project site [26].

### Sequence randomization algorithm (SRI-generator)

There are several possible approaches for randomizing nucleotide sequences while maintaining their N-mer oligonucleotide frequency composition. The simplest approach would be to randomly choose N-mer oligonucleotides based on their frequency composition and tile them one after the other. However, this approach does not necessarily preserve the frequencies of shorter length oligonucleotides that one may observe in natural sequences. For example, the random concatenation of N-mers as tiles artificially introduces dinucleotide composition bias created from the border of two adjacent oligonucleotide tiles – producing an overrepresentation of CpG dinucleotides and the like that do not match the SRI natural sequences. Therefore we chose a different approach which generates a randomized sequence one nucleotide at a time moving in a 5' to 3' direction.

We generate randomized sequences in the following manner. First we choose the largest oligonucleotide size (N) that is sufficiently sampled. In practice, this means avoiding sizes for which some of the oligonucleotides are never encountered in the input sequence (i.e. occur with zero frequency). Throughout our study we used 4-mer oligonucleotides (N = 4) because they were consistently well sampled across all of our input sequences, including a single large intron in the Figure 2C. The starting oligonucleotide is chosen at random, abiding by the frequency table for oligonucleotides of the chosen size (N). Next, we observe the last (N-1) bases of our sequence, and append a base to the 3' end, following the N-mer oligonucleotide frequencies. For example, if N = 4 and GTC were the last three bases in the growing random sequence, the frequencies of GTCA, GTCT, GTCC, and GTCG would be used in randomly adding the next base. For instance, suppose these four oligomers have relative frequencies of 0.5, 0.1, 0.2, and 0.2, respectively. Then the random number generator will append 'A' with a probability of 0.5, 'T' with a probability of 0.1, 'C' with a probability of 0.2, and 'G' with probability of 0.2. This final step is then repeated until the randomized sequence reaches the length of the input sequence. In contrast to the tiling method, our approach preserves the frequencies of short oligonucleotides in addition to preserving the N-mer frequency composition.

Finally, we made our SRI-generator work properly with sequences that have masked repetitive elements (where all sequences of DNA repeats are replaced by N's by the RepeatMasker program). Any non-A, T, C, or G bases are copied from the source sequence over the output sequence. The random sequences thus contain the same number of N's (or other non-A, T, C, or G bases) in the same positions as in the natural sequences provided as input.

The pseudocode for SRI-generator is presented in Additional file 2, while the source code (written in Perl) is freely available from our project website [26].

### CDS-generator

Several sophisticated algorithms are already available for the randomization of coding sequences [37,38]. However, here we used our own randomization approach developed by AF in 2001 while working on a context-dependent codon bias project in the Walter Gilbert lab [29]. We stayed with our program because we are familiar with the peculiarities of this type of randomization. In addition our approach gets the dicodon distribution of randomized sequences very close to that of the natural CDS.

### Program notes

1) We observe a gradual diminution of the difference between real and randomized sequences when using progressively larger oligonucleotides with the randomized sequence generation programs (SRI-generator and MRI-generator). The difference is not considerable, but it is noticeable. Therefore, we recommend the use of longer oligonucleotides in the construction of randomized sequences – to maximize the retention of short-range inhomogeneity – as long as the rarest oligonucleotide in the corresponding frequency table occurs at least ten times. We use tetramer frequency tables throughout the manuscript for the sake of consistency and since they can safely be used for analyses of individual loci having as little as 100 kb.

WARNING: In MRI-generator it is easy to shift the nucleotide content level of generated sequences by using thresholds that do not balance the number of content-rich and content-poor regions. One must experiment with the thresholds and use SRI-analyzer to confirm that the content of the MRI-generated sequence approximates that of the source sequence.

2) The graphical output provided with the online version of MRI-analyzer serves only as a quick visual aid. The true output is represented by large tab-delimited files containing a record for each window in the analysis. Each record contains flags indicating a content-rich or content-poor window and, for those records where one of the thresholds has been crossed, the corresponding sequence.

3) All programs are written in Perl and may be freely downloaded from the website. They are licensed under version 3 of the GNU General Public License (GPL).

4) The RNALfold program from version 1.6.1 of the Vienna RNA package was utilized locally on our computers with default parameters.

### Source for gene sample set

Our sample of 11,315 non-redundant human genes (with < 50% sequence identities between each other) was obtained from the human Exon-Intron Database, release 35p1 [39]. Samples of intergenic regions were obtained from Genbank human genome files build 36 based on the records from the Feature Tables. We used only plus strands for calculations because there are only fluctuation differences between plus and minus strands in the non-coding regions of mammalian genomes. Also, plus and minus strands have the same G+C and A+T compositions. All these samples are available from our departmental project site [26].

## Abbreviations

SS: secondary structure(s); MRI: mid-range inhomogeneity; SRI: short-range inhomogeneity; UTR: untranslated region; CDS: coding sequence(s); nt: nucleotide(s); kb: kilobase(s).

## Authors' contributions

JMB, TW, TD, JS, SA, SKR, SS were responsible for computational processing of all datasets and creation of all described programs. JMB and SS also created the Genomic MRI web resource. AF supervised the project, provided guidance and wrote the draft. All authors have read and approved the final manuscript.

## Supplementary Material

Additional file 1 Complete oligonucleotide frequency table for natural 5'-UTRs of 11,315 human genes and two SRI-generated counterpart sequences. This file is also available from our departmental project web page [26]. Click here for file

Additional file 2 Pseudocode and further explanation of the algorithm used in SRI-generator. This file is also available from our departmental project web page [26]. Click here for file

Additional file 3 **Distribution of strong local SS with respect to folding energy in masked intergenic regions of human chromosome 17**. Number of structures was measured within 1 kcal/mol intervals and normalized by 1,000 nucleotides of analyzed sequences. Results are shown for intergenic regions from chromosome 17 (red) and two independent SRI-generated sequences (gray). All sequences are masked with the RepeatMasker program. **(A) **Results with masked positions ('N's) retained, which reduces the density of secondary structures; **(B) **results with masked positions ('N's) removed in order to compare with the graphs in Figure 2. Click here for file

Additional file 4 **Comparison of MRI-analyses of GC-, AG- and GT-content with a 50 nt window in masked DMD intron 1**. The first intron of the *DMD *gene was masked using the RepeatMasker program. SRI-generated counterpart sequences retain all masked positions. In each figure the MRI pattern for the natural sequence and the randomized counterpart is shown above and below, respectively: **(A) **analyzed for MRI in GC-composition; **(B) **analysis for MRI in AG-composition; **(C) **analysis for MRI in GT-composition. Click here for file
