# Supplementary material for: Genomic mid-range inhomogeneity correlates with an abundance of RNA secondary structures
Source: BMC Genomics. 2008 Jun 12;9:284. doi: 10.1186/1471-2164-9-284 (PMC2442090; doi:10.1186/1471-2164-9-284)
Supplement: Additional file 2 — Pseudocode and further explanation of the algorithm used in SRI-generator. This file is also available from our departmental project web page [26]. [file 1471-2164-9-284-S2.pdf]

Algorithm 1 SRI Generator

Given: We define $\mathbb{B} = (A, T, C, G) = (\mathbb{B}_i)_{i=1}^{4}$ to be the sequence of canonical bases. Furthermore, let $\mathbb{O}^K = (\mathbb{O}_i^K)_{i=1}^{4^K}$ be the sequence of *oligomers* of length K, e.g., $\mathbb{O}^3 = (AAA, AAT, \ldots, CGG, GGG)$. (Notice that $\mathbb{O}^3$ has $4^3 = 64$ elements.) Similarly, let $\mathbb{F}^K = (\mathbb{F}_i^K)_{i=1}^{4^K}$ be the sequence of oligomer *frequencies* of length K, such that $\sum_{i=1}^{4^K} \mathbb{F}_i^K = 1$ and $\mathbb{F}_i^K \in [0,1]\ \forall i$. For each fixed i, the oligomer $\mathbb{O}_i^K$ has one corresponding frequency value $\mathbb{F}_i^K$. These frequencies are computed from a sample input sequence using SRI Analyzer. Let $\mathcal{N} \geq 1$ be the user-chosen oligomer length for which the frequency composition is being approximated. Finally, we assume the input sequence is composed purely of A, T, C, and G. For a modified version of this algorithm which handles impure samples, please see our source code.

Ensure: The output sequence(s) *approximates* the short-range inhomogeneity of the input sequence. In other words, the input and output sequence(s) will share a similar $\mathcal{N}$-mer frequency composition. The output sequence(s) will be *randomly* constructed to satisfy this constraint.

$\langle \mathbb{O}^{\mathcal{N}}, \mathbb{F}^{\mathcal{N}} \rangle$ = ReadCompositionTable() #Example for $\mathcal{N} = 1 : \langle\ (A, T, C, G), (0.25, 0.40, 0.20, 0.15)\ \rangle$
#Calculate the demarcation table for the random selection of the first oligomer.
$sum = 0$
for $i = 1$ to $4^{\mathcal{N}}$ **do**
    $sum \Leftarrow sum + \mathbb{F}_i^{\mathcal{N}}$
    #Let *partialSum* be an array such that $partialSum^{(i)} \in [0,1]$, for $1 \leq i \leq 4^{\mathcal{N}}$.
    $partialSum^{(i)} \Leftarrow sum$
end for
for each FASTA sequence, "*seq*" **do**
    $length$ = GetSequenceLength(seq)
    if $length \geq \mathcal{N}$ **then**
        $R_1 \Leftarrow$ GenerateRandomNumber$(0, 1)$ #Generate some random $R_1 \in [0, 1]$.
        for $i = 1$ to $4^{\mathcal{N}}$ **do**
            if $R_1 < partialSum^{(i)}$ **then**
                $randomSeq \Leftarrow \mathbb{O}_i^{\mathcal{N}}$
                Exit Loop.
            end if
        end for
        for $i = 1$ to $length - \mathcal{N}$ **do**
            $sum \Leftarrow 0$
            #The next base is chosen randomly using the frequencies of the 4 possible overlapping $\mathcal{N}$-mer sequence tails.
            $R_2 \Leftarrow$ GenerateRandomNumber$(0, 1)$
            $tail \Leftarrow$ Suffix($seq, \mathcal{N} - 1$) #Example: Suffix("hotdog", 3) = "dog".
            for $i = 1$ to 4 **do**
                $oligo \Leftarrow$ Concatenate($tail, \mathbb{B}_i$) #Example: Concatenate("hot", "dog") = "hotdog".
                $f \Leftarrow$ GetOligoFrequency($oligo$) #GetOligoFrequency($oligo$) = $\mathbb{F}_j^{\mathcal{N}}$ for one j such that $\mathbb{O}_j^{\mathcal{N}} = oligo$.
                $sum \Leftarrow sum + f$
                #Let *demarcation* be an array such that $demarcation^{(i)} \in [0,1]$, for $1 \leq i \leq 4$.
                $demarcation^{(i)} \Leftarrow sum$
                if $R_2 < demarcation^{(i)}$ **then**
                    $randSeq \Leftarrow$ Concatenate($randSeq, \mathbb{B}_i$)
                    Exit Loop.
                end if
            end for
            if $R_2 \geq sum$ **then**
                $randomBase \Leftarrow$ PickRandomBase() #Randomly choose a base from {A,T,C,G}.
                $randSeq \Leftarrow$ Concatenate($randSeq, randomBase$)
            end if
        end for
        WriteOutputFile($randSeq$)
    end if
end for
