# Supplementary figures and images for: Genomic mid-range inhomogeneity correlates with an abundance of RNA secondary structures
Source: BMC Genomics. 2008 Jun 12;9:284. doi: 10.1186/1471-2164-9-284 (PMC2442090; doi:10.1186/1471-2164-9-284)

### A Masked intergenic regions with N's

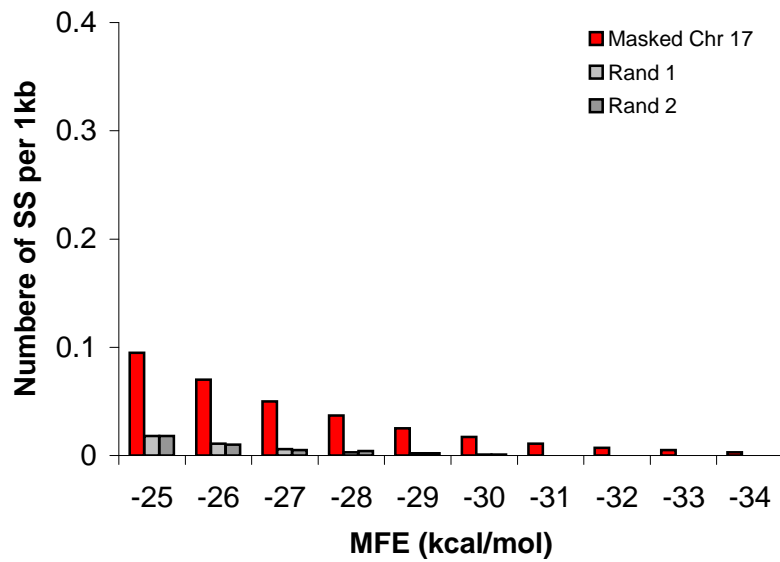

### B Masked intergenic regions without N's

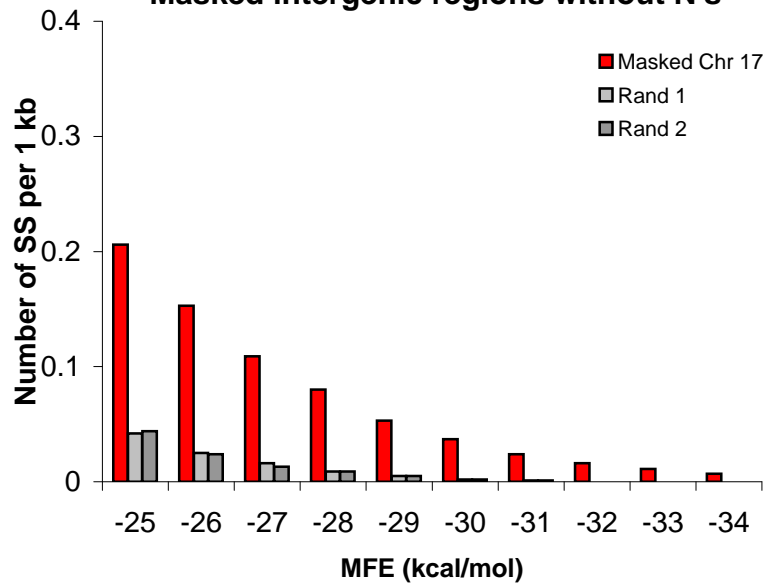

Supplement: Additional file 3 — Distribution of strong local SS with respect to folding energy in masked intergenic regions of human chromosome 17. Number of structures was measured within 1 kcal/mol intervals and normalized by 1,000 nucleotides of analyzed sequences. Results are shown for intergenic regions from chromosome 17 (red) and two independent SRI-generated sequences (gray). All sequences are masked with the RepeatMasker program. (A) Results with masked positions ('N's) retained, which reduces the density of secondary structures; (B) results with masked positions ('N's) removed in order to compare with the graphs in Figure 2. [file 1471-2164-9-284-S3.pdf]

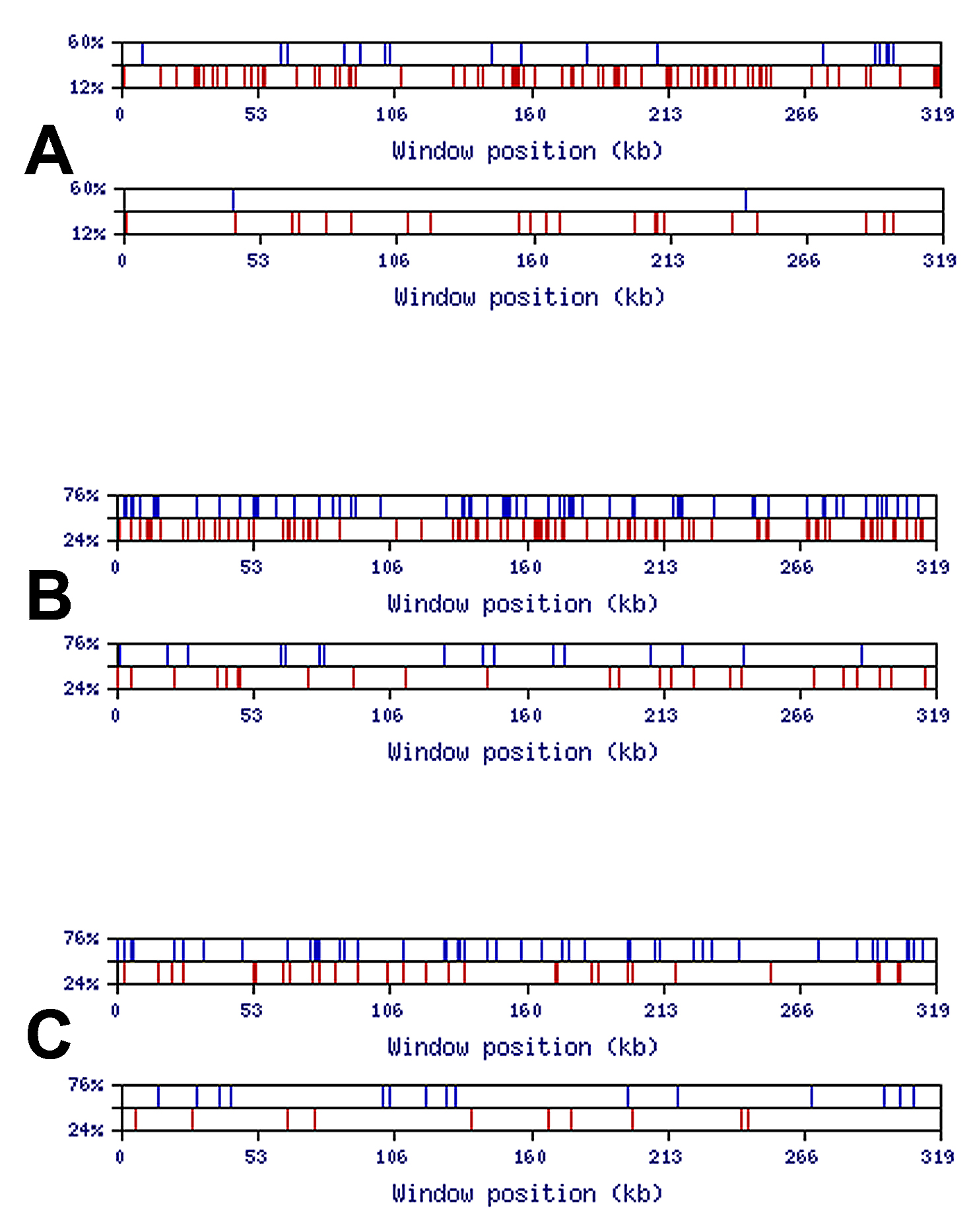

Supplement: Additional file 4 — Comparison of MRI-analyses of GC-, AG- and GT-content with a 50 nt window in masked DMD intron 1. The first intron of the DMD gene was masked using the RepeatMasker program. SRI-generated counterpart sequences retain all masked positions. In each figure the MRI pattern for the natural sequence and the randomized counterpart is shown above and below, respectively: (A) analyzed for MRI in GC-composition; (B) analysis for MRI in AG-composition; (C) analysis for MRI in GT-composition. [file 1471-2164-9-284-S4.jpeg]
